# *'Small small quarrels bring about happiness or love in the relationships’*: Exploring community perceptions and gendered norms contributing to male perpetrated intimate partner violence in the Central Region of Ghana

**DOI:** 10.1371/journal.pone.0225296

**Published:** 2019-11-21

**Authors:** Phyllis Dako-Gyeke, Adolphina Addoley Addo-Lartey, Deda Ogum Alangea, Yandisa Sikweyiya, Esnat Dorothy Chirwa, Dorcas Coker-Appiah, Rachel Jewkes, Richard Mawuena Kofi Adanu

**Affiliations:** 1 Department of Social and Behavioral Sciences, School of Public Health, University of Ghana, Accra, Ghana; 2 Department of Epidemiology and Disease Control, School of Public Health, University of Ghana, Accra, Ghana; 3 Department of Population, Family and Reproductive Health, School of Public Health, University of Ghana, Accra, Ghana; 4 Gender and Health Research Unit, South African Medical Research Council, Pretoria, South Africa; 5 Gender Studies and Human Rights Documentation Centre, Accra, Ghana; USC Keck School of Medicine, Institute for Global Health, UNITED STATES

## Abstract

In this paper, we explore gender norms held by men and women that might contribute to male perpetration of intimate partner violence (IPV) in Ghana. This qualitative research was conducted at the pre-intervention stage of a cluster randomized controlled trial. Our intervention uses community-based action teams to change social norms on gender and violence. Focus group discussions and in-depth interviews were conducted within communities. We found that male perpetrated IPV is a common phenomenon within the study communities, yet it is complex and experienced differently depending on the context. A woman’s non-compliance with gender norms provided context for the male partner to enforce societal conformity through IPV. Also, male partners’ misbehavior (e.g. alcohol abuse) may exacerbate IPV. Whereas the former is socially acceptable, the latter may be contested. Victims may challenge/counteract IPV using varying tactics (e.g. threats), which were mainly directed toward male partners’ immoral behavior. We conclude that there is a need to assess IPV with key considerations for female agency, as some victims may respond with violence. Moreover, some communities have the tendency to demonstrate more gender-equitable attitudes regarding male perpetration of IPV, as indicated by laws instituted by some traditional leaders to deter perpetrators. These are key learnings that can inform the design and delivery of various interventions that seek to address IPV.

## Introduction

Eliminating violence against women (VAW) requires legal and legislative initiatives coupled with community-based interventions, which target social norms [1]. Violence against women is an act of gender-based violence that results in or is likely to result in, physical, sexual or psychological harm or suffering to women [2] and IPV is the commonest form of VAW [3]. Globally, the prevalence of lifetime experience of VAW among women and committed by intimate partners ranges between 15–71% [4]. Since the 1990s, African women have particularly been vulnerable considering that one-quarter of these women in African countries report lifetime IPV prevalence of at least 50% [5,6]. In Ghana, it was reported that 92% and 34% of women experience sexual and psychological violence, respectively, from their partners [7].

The ecological framework explains IPV as a multifaceted involving individual, personal relations, community, and societal factors. At societal level IPV may be shaped by, law, policy, social norms and power relations which contribute to the public’s understanding and practical responses [8,9]. This is closely related to the community level, which focuses on context (e.g. unemployment and mobility). Also, personal relationships with family, friends, intimate partners and peers may influence the risks of becoming a victim or perpetrator of violence. At a lower layer individual considers personal histories and biological factors, which influence how individuals behave and or may increase their likelihood of becoming either a victim or a perpetrator of violence (e.g. victim of child maltreatment, psychological or personality disorders) [4].

This framework was adopted as the theoretical foundation for both research and the programming of the present intervention [8,9]. IPV in Ghana presents not only as an outcome of these multiple factors but also operates within a hierarchical structure of interactions. At the top of this hierarchy is a social structure of culture, religion, as well as the social organizations of kinship systems, which sustain societal inequalities in the form of patriarchal norms of male superiority as against female inferiority [10–12]. For these reasons a woman would endure abuse. More so, poverty and economic dependency sometimes, force women to stay in abusive relationships because they are totally dependent on male partners [10,12]. Unfortunately, women in the illiteracy and poverty brackets are in the highest risk group of IPV in Ghana [12]. However, there are instances where women are forced to divorce. This sometimes leads to regrets and stigmatization especially, when the church evokes open criticism of such individuals [13].

For the longest time, domestic violence cases in Ghana were treated as “private” matters that had to be settled at home. However, to curb forms of domestic violence, the Ghana government passed the Domestic Violence (DV) Act 732 in 2007, which mandates the Ministry of Gender, Children and Social Protection to fight domestic violence. Following the passage of the DV Act 732, the Domestic Violence and Victim Support Unit (DOVVSU) was established, and presently has offices in the capital cities of 10 regions and in most of the districts throughout the country. Despite the availability of these structures, if a woman is bold enough to report a case, she may be convinced by the family to withdraw the case.

The Sustainable Development Goal 5 (SDGs), which seeks to address gender equality and empowerment, also targets the elimination of violence against women and girls [1]. In this regard, the current trial titled ‘impact assessment of the rural response system to prevent violence against women’ was initiated in 2015 to evaluate the Gender Centre’s rural response system (RRS) in Ghana. The RRS uses community-based action teams (also known as COMBATs) to sensitize communities about IPV, whilst promoting a community-focused response mechanism. Here we specify a community as the setting for intervention [14]. As a setting “community” refers to a local geopolitical entity, within which members share some sense of identity or connection often defined by function, geography, common interests or characteristics, or by a combination of these domains [15–17]. However, it has been observed that communities may be locally diverse, exhibiting heterogeneities in religion, socio-economic status, educational background, interests, etc. [16] which may influence possibilities of IPV perpetration. This was demonstrated in Bangladesh, where men in more equitable communities were less violent and had more equitable attitudes about gender [18]. In this paper, we use qualitative methods to explore gender norms held by men and women that might contribute to male perpetration of IPV in four districts in the Central Region, Ghana. We illustrate the dynamics of IPV as experienced by victims and perpetrators, as well as highlight how IPV is legitimized through the performance of gendered norms by men and women, albeit differently. In addition, we identify various social mechanisms through which the act of IPV may be challenged.

## Materials and methods

### Study design

The qualitative data used for this paper was part of the baseline study for a larger randomized controlled trial that is assessing community-level impact of the Rural Response System (RRS) intervention which uses Community Based Action Teams (COMBATs) to reduce and prevent VAW in Ghana [19]. The cross-sectional survey carried out baseline indicated that about 34% of respondents had experienced IPV in the past year, with 21% reporting sexual and or physical IPV [20]. This qualitative research was conducted with women and men who had been victims or perpetrators of violence (n = 40 in-depth interviews), women and men from the communities (n = 12 focus group discussions), and COMBAT members (n = 2 focus group discussions). The scopes of inquiry of the FGDs and IDIs were to understand community perceptions of violence and social norms on gender and how these drive male perpetrated IPV. Participants were asked several questions, including gendered roles, how decisions are taken, how, why and what forms of VAW are experienced, and whether VAW is allowed. We ensured that our qualitative research reporting met the consolidated criteria for reporting qualitative studies [21].

### Study area

The trial is being carried out in four [4] Districts located in the Central Region of Ghana. Two of these districts are along the coast while the other two districts are inland districts. The four districts in the study (each comprising about 10 communities) were selected based on operational and program considerations (i.e. whether previous intervention work on GBV had been carried out in those districts by the Gender Studies and Human Rights Documentation Centre (Gender Centre), which is the intervention delivery partners for this trial. The selection of districts was done using a census map of the Central Region that showed Inland and Coastal districts. After excluding some districts because previous intervention work on gender-based violence has been carried out in them; two inland and two coastal districts were then purposively selected as study sites. Designated sites for control or intervention were separated from each other by a geographical buffer (at least one districtwide) to reduce spillover. Participating communities selected in intervention and control districts had comparable socio-demographic characteristics that cut across both rural and peri-urban areas. Communities, similar to national population characteristics, were predominantly Christian and thus, no purposeful selection based on religion was made. Additional details about the study design and participants are provided in the study protocol [19] and registration available on clinical trial.gov (#NCT03237585).

Adult literacy rate in the region is about 50%, with more men being literate (70%) compared to women (46%) [22]. The region is predominantly Akan speaking (82%) and Fante is the indigenous dialect of most districts in the region. Agriculture (including fishing) is the main occupation and it employs more than two-thirds of the workforce in many districts. Fishing is concentrated mainly along with the coastal districts whereas cocoa and oil palm production is concentrated in the inland districts. Other agricultural enterprises are pineapple and grain production.

### Study participants

A total of eight [8] focus group discussions were conducted, each comprising 7–10 participants. The focus groups were made homogeneous with regard to the number of FGDs conducted with each sex by selecting four male-only groups and four female-only groups. Four out of the eight FGDs were conducted within the two intervention districts (i.e. two male groups and two female groups per district) while the other 4 homogenous FGDs were carried out in control districts (i.e. one male and one female group per district). In order to reach saturation we adopted the proposed sample size [23,24], indicating that saturation is reached for homogeneous groups for after 17 interviews, and for FGD’s, 8 groups. FGDs were organized for male and female participants below 35 years and above 35 years, separately. This allowed for the expression of gendered views within a comfortable and convenient environment without so much apprehension. Also, the staff was gender-matched, whereby male and female research assistants moderated male FGDs and female FGDs, respectively.

Two mixed-group FGDs were carried out with those that were expected to deliver the intervention, “Community Based Action Teams” (COMBAT), to examine gendered social norms and how VAW is perceived in their communities. Males and females were not separated in these discussions because COMBATS work in teams of two (one male and a female) per design of the intervention.

Respondents with IPV experiences (either victims or perpetrators) were identified during the baseline survey by research assistants who had been trained and primed to identify such persons [20]. They were followed-up and privately asked to confirm if they have had VAW experiences. Those who admitted to such experiences were invited to participate in the IDIs. All in-depth interviews (n = 40; 21 females and 19 males) were conducted within the intervention communities. These in-depth interviews were organized at private locations (e.g. home, quiet locations near homes or workplaces) to allow individuals the opportunity to share experiences without hesitation.

All the participants identified for the IDIs were willing to participate in this study (i.e. 100% response rate). On a few occasions, not all invited participants for the community FGDs showed up. Consequently, some sessions were made of 7–8 persons instead of 10. Reasons for non-participation included emergencies and work. There were no outright refusal/non-consent in this study.

### Characteristics of participants

In all, 165 community members were interviewed, most of whom were married or cohabitating, professed Christian faith, and had attained junior high school level of education (grade 7–9). Few of the informants had lived less than 5 years in the community while most had lived there for over 16 years. Table 1 provides details of the socio-demographic characteristics of participants.

**Table 1:** Socio-demographic characteristics of participants.

Characteristic	Individual IDI		Community FGD		COMBAT FGD
	Female (n = 21)	Male (n = 19)	Female (n = 52)	Male (n = 53)	Mixed Group (n = 20)
**Age (years)**					
< 20	0	2	5	0	0
20–29	11	4	20	15	6
30–39	6	5	11	20	5
40–49	4	3	16	7	5
≥ 50	0	5	0	11	4
**Marital Status**					
Single	5	3	12	6	1
Married/Co-habiting	16	15	36	28	17
Separated/ Divorced	0	1	2	18	2
Widowed	0	0	2	1	0
**Educational Status**					
None	6	1	6	4	4
Primary	1	7	17	9	2
JHS/Vocational	14	3	27	13	10
SHS/ MSLC	0	7	2	17	1
Tertiary	0	1	0	10	3
**Religious Background**					
Christian	21	17	48	50	18
Muslim	0	2	3	3	2
None	0	0	1	0	0
**Number of years living in Community (years)**					
≤ 5	6	0	8	9	2
6–15	3	9	12	5	3
≥ 16	12	10	32	39	15

IDI–In-depth Interview, KII–Key Informant Interview, FGD–Focus Group Discussion. JHS–Junior high school; SHS—Senior high school; MSLC—Middle school leaving certificate

### Data collection

All qualitative interviews were carried out from May-June 2016. The scope of the interview guides were developed based on the primary and secondary outcomes of the RRS trial [19]. The interview guides used for the FGDs and IDIs have been included as supplementary files in the present report. Interview guides were translated into local dialects (Fante and Twi) by an independent consultant and then edited by bi-lingual members of the project team at the University of Ghana. The revised translations were independently back translated by another consultant who had not seen the English guides. The project team then used a consensus-building translation approach to finalize the translated interview guides. This involved discussing and resolution of discrepancies together with field research assistants during baseline training. The interview guides were subsequently pretested for clarity and suitability in a population with similar characteristics as the study population prior to the main data collection period.

Each FGD comprised 7–10 participants, led by a trained facilitator, supported by a note taker and lasted for about 1-hour 45 minutes. Interviews and discussions were organized at private locations away from the community centres. Participants were served snacks and beverage (malt) during the interviews and each participant was given an amount of Ghc.20–30 (the equivalent of USD $5–8) as transportation allowance.

For the IDIs, participants were interviewed in a secluded part of their compound or away from their residence (if desired) to ensure privacy during interviews. Thus, interviews were conducted with participants alone with no interference from their partners, children, and other adults. Interviews lasted about an hour and participants were given a beverage (malt) and water as well as Ghs 10 (~2.5 USD) as a token of appreciation and reimbursement for their time participating in the interview. All interviews were recorded both in writing and audio taping with the consent of participants. To ensure confidentiality, pseudonyms were used for participants during interviews.

### Data processing and analysis

All recorded information from the field were transcribed verbatim and included in our analysis. Transcriptions were augmented with researcher’s field notes made through observations and during FGDs and IDIs. An initial through reading through the transcripts and notes was preliminary in the development of the codebook. Various codes were identified and labelled in the codebook. The code definitions, as well as what quotes could or could not be categorized under each code were outlined, and examples to guide these decisions were also suggested. The codes were used to develop a codebook/manual by the qualitative research consultant. Following this stage, a team of four researchers and six research assistants met to review and test the applicability of the codebook using the raw data from the transcripts, which led to the expansion of codes.

Research assistants applied the codebook to sort and categorize all data using Nvivo version 11, and the first step towards this consisted of importing the transcribed data into the analytical software. The codes from the codebook were then applied as nodes in the software and the transcripts were studied one at a time to select relevant quotes and code them under appropriate nodes. Employing the constant comparative method of theme generation [25]. Several themes that cut across the various codes, as well as different kinds of datasets, were identified and corroborated among the entire research team. We report of no deviations from the research protocol with regard to the data collection in the field or in the planned analysis.

### Themes explored in FGDs and IDIs

The community FGDs and IDIs explored knowledge and perceptions about VAW, gender attitudes, women’s subordination, tolerance of VAW, social stigma for the victims of VAW, women’s involvement in household decision making, etc. FGDs conducted with COMBAT members’ explored themes on attitudes of service providers (state institutions mandated to handle VAW), and knowledge of community members on the impact of VAW.

### Ethical considerations

This research included sensitive topics that could potentially expose women to re-victimization if they had prior VAW experiences. Therefore, the study was designed to ensure that participants are not exposed to more than minimal risk of violence (more than everyday risk). All project staff received training on gender, gender-based violence, and research ethics prior to implementing the trial. We ensured complete privacy and the security of participants and researchers because asking about or reporting violence, especially in households where the perpetrator may be present at the time of the interview, carries the risk of further violence. Appropriate informed consent was provided in participants’ language of preference (English or local dialects). All prospective participants were informed about the purpose, risk, and benefits and that participation was voluntary. They were also informed that they may withdraw at any stage or skip any question in the research with no adverse consequences to them. All participants were assured that the information provided will be handled confidentially and that findings will be reported with complete anonymity.

Although we did not encounter situations where participants demonstrated distress or report being emotionally impacted by the research questions or intervention, our protocol had a referral provision for such instances. Interviewers were provided with a nationwide list of offices and contact information for the Domestic Violence and Victim Support Unit (DOVVSU) and trained to advise participants that they can seek help from the Probation and Social Welfare Officer at the district level. The facilitators and research assistants were also trained to identify participants who are visibly distressed during the interviews or intervention sessions and with their permission refer them to the appropriate state agency.

A written informed consent (this was achieved via thump-printing for illiterate participants) was solicited from all participants before participation in this study. Ethical approval for the study was secured from the Institutional Review Board at the Noguchi Memorial Institute for Medical Research, University of Ghana, (CPN-006/15-16) and the South African Medical Research Council’s Ethics Committee (EC031-9/2015).

## Results and discussion

During the analysis, we explored community members’ perspectives of male perpetrated IPV within the study communities. Emerging themes such as IPV experiences and its complexities, dynamics of occurrences, and gendered interpretations, which either legitimize or delegitimize IPV practices are presented here.

### Contextual dynamics of IPV forms experienced

In preparing “The World’s Women,” the United Nations Statistics Division undertook a compilation of data collected through surveys (1995–2014) addressing violence against women. This report indicated that in all societies, to varying degrees, women are subjected to physical, sexual and psychological forms of violence [3,26,27]. However, based on available data reported prevalence of IPV showed that prevalence were generally higher within the African region compared to Asia, Latin America, the Caribbean and Oceania [28]. In this light, it emerged in our study that male perpetrated IPV was a common recurring practice. Although the co-occurrence of IPV forms has been reported previously [29–31], community members also described these practices as being cyclical in manner, which may lead to complex and possible interactional sequences associated with the violence [32]. In Ghana, specifically, it has been reported that multiple forms and types of violence are experienced [33]. Community members mentioned diverse forms of IPV (verbal, sexual, physical and financial); these were often described as a continuum of male practices with differing consequences on women and children. For example, a female (40–49 y/o) victim of IPV said that *“yeah*, *my husband is able to go behind me and go in for another woman and when I talk about it the two of them become one and insult me…But because of this same womanizing issue*, *the last time he strangled me*.*”* In this story, she would experience insults first, followed by beatings; whereas another victim said she would experience financial abuse, which is followed by fights:

“*as I said*, *it was because of money*, *when I ask him for something*, *he will tell me he doesn’t have*, *and when I continue to say it we end up fighting*.*” (20–29 y/o*, *Female*, *IDI)*

Scholars argue that a myriad of tactics may be used by men to exert control and authority over female intimate partners [34–36]. These tactics may simultaneously embrace economic relations, power relations, and affective relations [37]. This becomes more pronounced within contexts where there is female dependence due to widespread poverty [12,38]. The Domestic Violence study conducted in Ghana reported that the most common form of domestic violence reported by women was financial violence (13%) [33]. It is known that, although IPV occurs across all socio-economic strata, women living in poverty are the most severely affected [39].

### Financial IPV

Other examples of female dependence and male partners’ irresponsible behavior and disregard for family needs emerged in our data. Unlike other forms, female victims linked financial IPV with the children and family economy. They noted that the male partners’ disregard for their responsibilities negatively affects both the woman and children. For instance, one female victim recalled that she would cry and be in a state of despair since she does not have an alternative source of income:

*“Women struggle so much*. *The men*, *when they have children with you*, *they do not take care of them*, *after having children with you*, *they go for another woman and have children with her too*. *In this case*, *it is a bother*. *Me for instance*, *I would cry*. *Sometimes I weep*, *I go and sit somewhere and cry the time away because the man does not mind us*. *I do not work or learn a vocation*, *I don’t do anything so if I don’t beg for GH 0*.*20p*, *I would not even get money for water*. *It is very painful if the man does not take care of you*.*”* (20–29 y/o, Female, FGD)

In the case of another female victim, her husband would take care of the children, but neglect her needs. This paints a picture where the woman and the children are totally dependent on the man for their economic survival;

*“No*, *I don’t know what I have done to him*, *because he will not talk to me or mind me*. *He will not give me food to eat but rather he will eat and sleep*. *As for the children if he gets some he will give them*, *but me the grown-up person he will not mind*.*”* (20–29 y/o, Female, IDI)

In another instance, an older male community member highlighted how some men may not attend to the financial needs of his wife, especially when she is not working and totally dependent on the man:

*“…maybe when the woman gets a funeral and the family gives the woman a token to pay in order to support*, *but because she's not working she doesn't have money so the man has to help her*. *In my area it happens a lot*, *the man can say that he's not from the same family with her so she should find a way to solve her problem*, *even ghc10 he won't give her*.*”*

(40–49 y/o, Male, FGD)

### Verbal IPV

Similar to financial abuse, verbal IPV presents as an extension of female dependence within patriarchal societies [33]. Within these contexts, gendered inequalities may be normalized through coercive mechanisms which permit men to dehumanize women and treat them as their own property [11,12,31,38,40,41]. Unfortunately, women are often seen, less as independent individuals and more as sex objects, which has implications for violent behavior directed toward them [42]. It is argued that the extent to which men objectify women is positively associated with their likelihood they will abuse their female intimate partners [42].

For instance, participants shared instances where insults from a male partner commodified his female partner as an object he regrets having acquired and invested in. Also, in another instance, the male partner likened his wife to a dog (something that can be owned). In both cases, the female partner was dehumanized:

*“One man has been quarrelling with his wife and saying hurtful things like*, *‘If I knew you would behave this way in marriage*, *I should have rather bought a goat and reared it*.*’ He even added that if he had bought the goat*, *by now*, *it would have brought forth a lot of offspring and given him so much more profit than marrying her*. *This is just because the woman has not been able to bear him a child yet*.*”* (30–39 y/o, Male, FGD)

*“Just three days ago*, *I was there when a man in my area was quarrelling with his wife over a trivial issue*. *He insulted her*, *called her a cheat*, *a dog and all sorts of things a man should not say to a woman*.*”* (30–39 y/o, Male, FGD)

### Sexual IPV

It has been argued that since men are socialized to be the head of the family in Ghana, by default decision-making authority regarding reproductive behavior including sexual encounters are deferred to them [38,43]. Although marital rape is not explicitly stated as a crime in Ghana, it is implied in the Domestic Violence Act which was passed in 2007. A study on perceptions on the criminalization of marital rape in Ghana showed that that only 3% of married men did consider non-consensual sexual acts in marriage as rape as against 18% of women [44]. However, the cultural context adequately provides a belief system that helps to maintain the existing gender relational structures [11], leading to either sexual or physical abuses of women. Comparatively, few sexual violence situations were described, and mainly from male perspectives. Also, we noted that male perpetrators of these acts of abuse showed remorse. For instance, a male perpetrator shared an experience when he forced himself on his female partner. He mentioned that this act resulted from the female partners’ disregard for his sexual advances. He described a situation where he had to physically overpower her. But he became remorseful after the event:

“*Oh*, *I force her*. *I told her*, *chaley (friend) today I feel oo so I want to have sex with her but she says chaley today am tired but I said oo what are you talking about*, *that is why I told you I am In love with you*, *you are my girl so you have to allow me to do it but as I said I had to force her …*.*I didn’t want to do it but after I force her to put my this thing inside she was very calm but after I finish she was so sad so I was also disappointed to force to have sex with her*.*”* (30–39 y/o, Male, IDI)

Another male perpetrator shared a similar experience. According to him after forcing himself on his female partner he also looks out for some signs, which will require that he will go to the partner and render an apology to her:

***‘‘****the effect is seen is for instance if I forcefully have sex with my lady*, *sometimes when I am about to have my shower afterward*, *there is this particular water we the Muslims use to bath*, *I notice her pubic hair on my manhood*, *immediately I will return and come and apologize to her to forgive for what I did*.*”* (30–39 y/o, Male, IDI)

Also within a focus group discussion, an elderly man shared similar sentiments of how he forces himself of his wife in instances when he is drunk:

*“The fact is that*, *maybe I travelled and when I returned I was a bit drunk and smelling of alcohol so if I go close to her*, *she will say no*, *but me too once I have the urge or desire*, *I will force myself but she will also not want to allow me*. *So right there on the bed*, *you will see that there will be no peace because I would also insist on doing it*. *So that is where the misunderstanding will start from*, *I will force my way through and make sure I have that sex with her before I go to sleep*.*”* (≥ 50 y/o, Male, FGD)

### Physical IPV

Physical IPV is often driven by traditional cultural framings within the larger society that places men as heads of households, whilst endorsing physical forms of violence [30]. Within this context, men are socialized into exerting authority through physical abuse (beatings, punches, dragging, kicks, slaps, etc.). For instance, a female victim described a situation where she was beaten and dragged by her male partner during pregnancy. She mentioned that the partner would then run away after physically abusing her. Another victim mentioned her partner used a belt to whip her during pregnancy:

*“Ei*, *he could beat me and even drag me on the floor*. *It was my uncles who came to get me*. *I was pregnant at that time and he used to beat me*. *He could really beat me and drag me on the floor and when he is done he would run*.*”* (30–39 y/o, Female, IDI)

*“He was beating me when I was pregnant*, *one day he beat me with the belt and it left a mark on my stomach*. *When I gave birth to the child the mark was on the forehead of the baby*. *When his girlfriend provokes me*, *and I also retaliate back then he will beat me for insulting his girlfriend*.*”* (20–29 y/o, Female, IDI)

There was a similar situation which an elderly man within a focus group discussion shared as something which he witnessed within his community:

“*There was a certain guy who use to live in the last bungalow over there and he had the two kids with a lady…they were separated but the woman always came to visit the kids*. *So on one occasions the lady came to visit the children and I don’t know what happened the man was I mean beating her up so the lady had to run out and then I happen to be standing there so as the first person she saw*, *she ran towards me*. *The man was ‘charley’ aggressively pursuing her*.*”* (≥ 50 y/o, Male, FGD)

Similar to other African countries, Ghana is known to be a patriarchal society, which normalizes gendered inequalities through these forms of IPV [11,40,12,38,41,31]. Primarily, female dependence, especially in the context of widespread poverty culturally, may permit men to treat women as their own property and expect women to be submissive, and passive within marriages [12, 38]. Moreover, the cultural context of religion adequately provides a belief system that helps to maintain the existing gender relational structures [11]. For instance, the Bible is quoted to literally, suggest men as heads of the family and women as weaker vessels [45]. Also, in the traditional religious context, women may be put into religious bondage or enslavement to supposedly pay for the crimes of ancestors [11]. Within this context, women may be socialized into accepting forms of IPV. Besides these notions, there is other past research which argues that religion provides a rather than create a context for abuse [45]. There are interventions from both governmental and non-governmental sources and have aimed at both prevention and cure. However, such moves by women may be seen as terminal, leading to a divorce, which case women may not want that to go the length. This is because post-divorce stigma in Ghana can be extreme and women may feel regretful [46]. Women tend to be more concerned about others’ approval because of the logic that a person’s identity and self-worth are socially conferred or denied.

### Complexities of IPV experiences

Despite extensive experiences of varying forms of male perpetrated IPV, recent research demonstrated that IPV can be much more complex and may defy simplistic explanations and “binarisms” [35,47], especially regarding victimization behavior.

In acknowledging these diverse arguments, caution must be taken to critically examine accounts of violent behaviors [48]. Within our study communities, one male participant acknowledged committing violence against his female partner. According to him, the female intimate partner-initiated violence by holding his hand and placing it on her cheek, whilst challenging him with threats of reporting. He mentioned that he decided to respond with violence. Even though his account depicts violence as produced through actions by both partners, it also demonstrates alternative ways through which his female partner could challenge the man’s violent behaviors by using threats:

*“There are times the man is not ready to lift his hands at the woman*, *but the woman keeps daring them to try if they are men enough*. *Ahhhh*, *I’ve done it before*! *She*, *took my hand… yes*, *she picked my hand and placed it on her cheek*, *and she told me if I was man enough*, *I should slap her*. *I decided to slap her just to see what would happen*. *The women keep threatening the men that if they dare touch them*, *they would let the police arrest them*.*”*

(≥ 50 y/o, Male, COMBAT, FGD)

In a similar account, a male COMBAT shared an instance of a frequent IPV situation that he was confronted with. He recalled that the woman, in this case, is strong and sometimes responds to her male partner’s abuses with violence. This demonstrates that although the man may initiate violence, the female partner can also counteract his behavior by responding with violence:

*“The man is beating the woman*. *Every single day*, *they bring the issue to me…every week*, *their fights get bloody*. *The woman is strong…and the man is also in his twenties*. *At times*, *the woman beats the man*, *other times*, *the man wins the fights*.*”* (≥ 50 y/o Male, COMBAT, FGD)

These findings may be examples of conceptualizations of women’s agency in relation to IPV [47,49,50]. In such an interpersonal relationship, we apply a more dynamic and interdependent framing of agency, by considering the observed actions of partners, meaning, motivation and purpose which they bring to their actions [49,51]. We believe that these women were negotiating agency with their male partners. In one instance, it was through open defiance as she challenged the male partner. In another instance, it was through the use of more indirect strategies, such as manipulation [49]. The use of strategies ranging from moments of defiance to forms of bargaining, negotiation, manipulation, and resistance emphasizes the multiplicity of women’s resistance and resilience strategies, especially within contexts were individual choices and actions are constrained by societal norms [45,47,49,51,52].

### Acceptance of male perpetrated IPV for enforcing female gendered roles

Perspectives on the social justification of IPV have been presented by various scholars [5,53]. Studies in Africa and Ghana especially have reported the societal endorsement of male perpetrated IPV [11,31,40], noting that women were more likely to justify IPV than men. Within this context, females accept that their disobedience and disrespect regarding domestic and sexual roles are likely to attract a disciplinary measure, which may be in any IPV form [5]. This is often observed as a gendered mechanism, which targets female compliance, mainly. Consequently, we found consistent patterns relating to who is responsible for disciplining and what measures are employed. The nature of this enforcement mechanism creates and maintains communal structures and enactment of social practices (housework, paid labor, and child-rearing), which may underlie IPV cases [37].

The Ghana Domestic Violence study in 2016 reported that women were more likely than men to find wife-beating acceptable and also blame women for rape if they wore revealing clothes [54]. These findings suggest that some forms of domestic violence were still considered acceptable in Ghana and that domestic violence in Ghana may persist due to harmful social norms that made women more accepting of abusive relationships. Also, findings from the Domestic Violence study pointed to the ‘expected’ submissiveness of women within conjugal relationships, women’s roles as primary caregivers (for children) and their economic dependence on men as reasons for them to accept violence. For example, within our study communities, female partners are expected to diligently play domestic (e.g. cooking and washing), and reproductive (e.g. childbirth and caring for children) roles. They must also live up to expectations of being respectful and submissive to their male partners. In this case, when asked generally about women’s lives in the study community, a woman (40–49 y/o) mentioned, that “*the women go to the farm early in the morning and come back in the evening*”. Despite this long day of work, women are also expected to care for their husbands. As one participant said, “*You have to cook for your husband to take to the farm and also fetch water for him to bath*.” Another woman said, “*Women are supposed to sweep around and collect the rubbish*.”

Women in these communities accepted responsibility for these roles and used such gendered norms to legitimize male perpetrated IPV. Not only did they willingly suggest IPV as penalties for neglecting their duties, but they also willingly deferred the disciplinarian role to their male partners. For instance, a young woman mentioned during a focus group discussion for younger women, below 35 years of age, that if a woman shirks her roles then her male partner could punish her by refusing to give her money:

*“Please yes*. *Maybe if a woman shirks her responsibilities at home*, *she needs to be disciplined*. *The man can refuse to give her money as punishment*.*”* (20–29 y/o, Female, FGD)

Also, another woman during a focus group discussion for women above 35 years of age, mentioned a female partners’ disrespectful attitude can attract financial IPV as a form of punishment. For her, this may be done to deter women from being disrespectful and quiet them:

*“Please if a woman is disrespectful*, *she deserves to be punished by the husband*. *If she is punished for a while*, *she will stop…If a lady is disrespectful*, *a man can refuse to give you housekeeping money*. *After a while*, *she will be quiet and not disrespect*. *This is one of the punishments*.*”* (30–39 y/o, Female, FGD)

Additionally, a victim shared an experience where she willingly accepted IPV as a punishment, even though she was sick. She mentioned that her husband upon returning home to find that she had followed a friend to their farm slapped her:

*“I have received some beatings before*, *and I accept my fault that what I did was not good and that was why I was beaten*. *I was sick and when my husband went to work*, *I felt a bit better*, *so I went with a friend to her cocoa farm not knowing he had asked for permission to leave work early because I was sick and when he came home*, *I had gone to the farm*. *When I came*, *he didn’t spare me*, *he slapped me I saw lighting and sparkles in my eyes*. *He warned everyone off not to invite me to their farm again*.*”* (40–49 y/o, Female, FGD)

Moreover, female insubordination demonstrated through refusal to engage in sex with the male partner may attract IPV. Refusal of sex is often taken seriously, as it may involve a perceived threat to the relationship, or alternatively may relate to one partners’ desire to dominate or exert power over the other partner [55]. For instance, a woman shared experience within the study communities about a lady who *“was in the earlier stages of her pregnancy and her husband wanted to sleep with her and she refused*. *Yes*, *he beat her up*. *He used a stool to hit her stomach*.*”* This notion was confirmed by a younger woman during an in-depth interview who mentioned that satisfying the male partner sexually, is a key female responsibility among other roles:

*“Mmm*, *I feel that you should do what the man wants for him*, *for example wash his clothes*, *and take care of his feeding*, *bathing*, *dressing; a woman should be able to help with those things between the man and herself and being able to give your husband sex is something a woman must do*, *if you don’t allow him and he forces you*, *you can’t say it is rape*.*”*

(20–29 y/o, Female, IDI).

### A position of male power to commit IPV

The unequal distribution of power within traditional African marriages, the impact of polygamy, and the acceptance of male promiscuity provide enough context for male perpetrated IPV. At play is gender power inequities, operating at both societal and interpersonal levels [56]. In Bangladesh, for instance, Yount and colleagues demonstrate, that both the gender norms in a man’s community and his exposure to violence during childhood are essential determinants of his likelihood to perpetrate IPV [18]. Processes of socialization and culture encourage such inequalities through its definition of what is normal and acceptable within patriarchal societies [57]. This leads to a celebration of ideals of masculinity, such as male strength and toughness, often displayed in the possession of multiple female partners. Although this power position for males translates into predatory sexual practices, and other acts of violence against women, it is endorsed, as was found by Jewkes and Colleagues in cohort studies conducted in South Africa [39,58,59]. This situation gives impetus to Gaventa’s (2006) description of invisible power; which shapes the psychological and ideological perceptions of violence against female partners [57]. Consequently, IPV may be kept from the minds and consciousness of different players involved, including those directly affected by the violence [57]. By influencing how individuals think about their place in the home and society, this level of power shapes people’s beliefs, sense of self and acceptance of the status quo (IPV)–even their own superiority or inferiority [51, 59, 60].

In this case, the wife is seen to be challenging her husband’s authority and prerogatives should she inquire about his extramarital involvements [61]. Previous studies suggest IPV as a social discourse, which maintains power hierarchies and inequalities, with men accorded headship of the family [11,31,37,38,43,62]. Often, this male authority extends to the entire household membership, including the female partner. Therefore, in our study, we realized that some women passively accepted IPV practices, whereas men claimed the status of power and the right to correct. Within this superior status, male perpetrators mentioned that they assumed the role of surveillance over female actions and inactions. These are aimed at enforcing their compliance with gender norms, just as they would do for a child. For instance, one male perpetrator mentioned that *“we sometimes beat women to check or correct their ways*, *but not for fun*.*”* In another interview a male perpetrator shared his experiences and mentioned why he caned her:

*“My second wife*, *I caned her*. *Yes*, *I used a cane to whip her*. *I came from the farm only to find my wife sleeping instead of joining me on the farm*. *Meanwhile*, *she wasn’t sick*. *This was the main reason why I caned her*.*”* (≥ 50 y/o, Male, IDI)

Another male perpetrator rationalized his use of physical IPV forms as a disciplining tool for drawing his partners’ attention to her subservient position within the family. He identified female partners’ negative attitude such as throwing her hands at him as acts which attract his wrath. He uses IPV to remind his female partner about the unequal power relations that exists within the home; demonstrating that these positions are irreversible:

*“Erm*, *it’s because she did something wrong that is why maybe I threw my hand and I am showing you that the way you are going is no correct and so I am putting you on the right way*. *Because there is no woman who will throw her hand to hit her husband and the husband will forgive her*. *And so*, *I need to make you understand that it’s because of me that is why you are in the house and so I am above you*. *And so once I am above you anything I say in the house you have to comply*.*”* (40–49 y/o, Male, IDI)

### IPV as a desirable interpersonal act

Additionally, our study, like previous research, suggests IPV as an interpersonal act, which may be used to communicate love, disapproval of specific behavior, or resolve conflict situations [55]. Obviously perceiving IPV as an interpersonal act places it within a domain of privacy, intimacy, and confidentiality; a context which does not easily lend itself to external scrutiny [32,43,62,63]. In their review, Flynn and Graham (2010) indicate that aggression may be used as a mode of communication [55]. In some instances, studies found that female partners may look for attention, or advancing personal interests through provocative behaviors, which may, in turn, attract male violence [64,65]. This is worrying since it is not a constructive means of interpersonal communication and is also suggestive of deficiencies within relationships.

Descriptions of IPV as an interpersonal act were mentioned only by male participants, and not female participants. Within focus group discussions conducted with the study communities, some men described IPV as an act, which is desirable among women. Some males claimed this stems from the belief that it is only a caring partner who will feel enraged enough to take any action when provoked. Also, such acts are expected to build the relationship and not destroy it:

*“Some of the women love to be beaten*. *The thing is*, *when they are beaten*, *they believe their husbands love them and afterward all the disputes are settled between them*.*”*

(30–39 y/o, Male, FGD)

“*There are some women who like beating*, *so she will do everything for the husband to beat her*. *There is a saying; small*, *small quarrels bring about happiness or love in the relationships*.*”* (20–29 y/o, Male, FGD)

### Beyond victim blaming: Moments of contesting male authority

Despite widespread IPV justifications among our participants, recent global arguments point to possible increases in rejection of IPV due to world society influences on individual attitudes [66]. These arguments are grounded in recent transnational advocacy and development programming on dimensions of VAW. Additionally, the world is observing the rapid dissemination of globalized norms about rejecting violence [66]. Pierotti (2013) in a review mentioned that the global promotion of norms provides moralized guides for assessing individual behavior, regardless of location. Therefore, women with greater access to global cultural scripts through urban living, education, and access to media are more likely to reject IPV [66]. Similar to this argument, our study found participants who judged male partners’ behavior ended up rejecting some forms of IPV. They blamed male partners’ misbehavior as the cause, whilst questioning their authority to discipline. For example, men were blamed for IPV cases related to their own infidelity, alcohol use /abuse, and financial difficulties.

Often, romantic relationships are predicated on sexual and intimate exclusivity, and when partners commit sexual or romantic infidelity, IPV often ensues [42]. In societies where polygamy is approved, women are often perceived as not having the right to question the husband’s extramarital affairs [36]. Due to changes in society, women may attempt to question the male partner’s extramarital affairs, which may lead to IPV. For instance, a woman within a study community, made mention of the fact that men resented being quizzed about their extramarital relationships and used violence as a defense mechanism to keep their female partners from interrogating them. For this participant, the woman cannot be blamed in such cases:

*“Sometimes all the blame is put on the women but mostly it isn’t so*, *if you have a partner whether husband or boyfriend and you hear something about him and you ask him he will respond first in anger and we women are not as patient as men so when he gets angry the women will also talk back and that will result in violence*. *So mostly the problems are from the men*.*”* (30–39 y/o, Female, FGD)

Moreover, alcohol use and abuse are known to be associated with greater perpetration of IPV [39,67,68]. In Ghana, and elsewhere, studies show a strong relationship between husband’s alcohol drinking behaviors and IPV [33,69,70]. Stimulants such as methamphetamine and cocaine are also consistently linked to the perpetration of IPV [68]. People use and abuse substances (e.g., alcohol, cocaine, marijuana) for many reasons, including the need to cope with relationship conflicts. But such self-medication can backfire, creating more relationship problems by spurring IPV.

For instance, a female victim mentioned that her male partner used to consume alcohol and other drugs, which made him violent and frequently picked quarrels with her on very trivial issues. When the male partner gets drunk, it was difficult for her to reason with him on anything, and this led to insults or a fight. A situation that even the man’s friends disapproved of. She rejected this misbehavior, by leaving the male partner:

*“Yes*, *it is not only alcohol*, *but he also smokes cigarettes*, *weed and the alcohol as well so…everyday fight*, *everyday fight…if I had stayed there*, *I would have been dead*. *He gets drunk*, *you see*? *So he makes an issue out of any trivial issue*. *Take the children’s issue for example; he says I should not leave the children to play outside*. *I cannot also ask our children not to go and play outside*. *So because of the weed and other things that he smokes he does not understand anything I would say*. *But he would keep insulting me and people will…all his friends will be angry with him*. *He will say to me*, *‘You are foolish*, *as for you*, *you are a fool*, *stupid person”*! (30–39 y/o, Female, IDI).

Furthermore, there was a rejection of male perpetrated IPV possibly driven by the man’s inability to financially support the family. Although poverty on the part of the female partner leads to IPV, it is also known to lead to IPV in situations where men are battling to live up to their socially constructed role as breadwinners [47]. This kind of violence becomes a norm, where men are violent against the female partners they can no longer support economically [39]. Such impulsive violent behavior, according to Gottfredson and Hirschi, is a key characteristic of people with low self- control [71]. In their failure to consider the negative consequences of their actions, perpetrators tend to engage in acts of violence. Although they posit that self-control is a trait, developed, and stabilized, early in life, Wikstroöm analyze self-control as a situational concept, by emphasizing on its complexities. Therefore, exercising self-control or otherwise are choices made when an individual responds to environmental stimuli. An individual will respond to different environmental stimuli with varying degrees of self- control, [72]. For instance, a male participant blamed the pride of men as a key element in IPV instances, which result from experiences of financial difficulties.

*“Another factor is also about eating and preparation of food in the house*, *it can also bring violence*. *Chop money can also be a factor in generating violence in the house*. *Maybe even if the man is financially unstable*, *he won’t even inform the woman of the fact that he doesn’t have money today but the moment the women ask money then he will use pride*. *Pride can never solve that problem*.*”* (≥ 50 y/o, Male, IDI)

### Condemning IPV as a conflict resolution mechanism

A few male participants perceived IPV as reprehensible and unfair. Specifically, excusing the use of IPV as an interpersonal conflict resolution strategy was condemned. The use of IPV as a conflict resolution strategy is worrying, considering the availability of several other non-violent resolution options (e.g. mediation, dialoguing, forgiveness, and reconciliation). Use of alternative conflict resolution methods by male partners was advised since physical violence is criminal and punishable by law:

*“It’s not good and no matter how the problem is you don’t have to beat your wife or girlfriend because you might even get arrested if things get out of hand so if I have a problem with my girlfriends what I normally do is that I go out to my friends or maybe go out to have fun or I will not come to the house at all*.*”* (20–29 y/o, Male, IDI)

### Community disapproval of IPV

Furthermore, IPV contestation may be found, either at the national or community levels. Increasing attention to gender violence as a global policy concern was spearheaded by international women’s movements, leading to the Vienna Declaration in 1993 and the United Nations Declaration on Violence Against Women [73]. Although feminist mobilization influenced the development of international standards and norms on gender-based violence, they have also become increasingly visible in national legislation [73]. This has compelled several countries to craft laws on domestic violence [74] and also be seen as a part of global entities, which are addressing this problem. In this regard, there has been a shift in attention from acts of individuals to the ways in which institutions, social structures, and communities produce as well as respond to violence.

In our study, we realized that IPV was not only disapproved at the interpersonal level but also by some community authorities. It was noted that in some communities all forms of violence, IPV inclusive, are openly condemned. They were perceived to be disruptive to a peaceful environment and punishable by local laws. Participants indicated that laws have been instituted by traditional leaders (chiefs) to deter perpetrators, and ultimately minimize IPV occurrences in the community. There are instances where the chief personally warns particular couples to desist from the act:

*“Our chief even came around someday to warn some married couples who had misunderstanding going on in their marriage*. *Since I came to this community*, *these things have been happening*, *so he (chief) even got furious to the extent of ordering the okyeame (Linguist) to make announcement and put a warning message across in regard to the rampant violence going on in the community so the elders advised some of the people who were involved and that they have to minimize those behaviors in order to bring peace into the community*.*”* (20–29 y/o, Male, IDI)

Similarly, we noted that there are instances where other community members may condemn IPV either directly or indirectly. A situation that clearly shows that IPV disapproval may not be openly expressed:

*“Yeah*, *in fact*, *generally we frown down upon this thing of violence behavior against women and any time a woman’s rights are violated you will notice that the people immediately around either rebuke directly the source of the violation or they will go behind scenes and say*, *‘Ooooh I think what you did wasn’t the right thing so please don’t let it happen again*.*’ So*, *in fact*, *it is not you know we don’t sanction it at all*. *I think we frown seriously on violence against women*.*”* (≥ 50 y/o, Male, IDI)

### Conclusions

The authors constitute a team of African women and men researchers, who are located within both academia and practice on the continent. As the lead person who conceptualized and initiated the writing of this paper, I refocus this positioning on myself. I am a Ghanaian who was born in Ghana and has lived within this context for the most part of my life as a lecturer and a researcher. Although I have no personal experience of intimate partner violence, I have often witnessed it in the most diverse forms in Ghana and elsewhere. With full knowledge that these previous observations may bias my perspective on this matter, we involved a larger team of experts with relevant skills in qualitative research, who were a part of this study from the data collection through to the analysis and presentation.

We used our study to illustrate the dynamics of IPV as experienced by victims and perpetrators and highlight how the act is legitimized through varying performances of gendered norms. However, we need to mention that this research included several sensitive topics regarding violence, and questions requiring victims to recount traumatic events, which possibly may have led to re-victimization and the risk of psychological distress. We acknowledge that such a situation may have discouraged participants from sharing full details of their experiences or may not have been in a sound frame of mind for accurate recollection. Despite these limitations, our findings advance the current understandings of male perpetrated IPV in Ghana. Like other studies, we found that IPV is a common phenomenon within the study communities [33]. However, we emphasize that it is complex and experienced differently depending on the context. Women disregarding conventional feminine gender roles (e.g. cooking, childbirth), experienced male perpetrated IPV (e.g. financial, physical). Also, male partners’ misbehavior (e.g. alcohol use/abuse) or financial instability may drive IPV. Regarding IPV complexities, we observed that whereas the man’s authority to use IPV to enforce conformity to gender norms was acceptable, the use of IPV in instances of male partner’s misbehavior may be contested. Female partners may accept, challenge or counteract IPV through different tactics (e.g. threats, violence). Additionally, communal attempts at IPV, often seek to address overt (e.g. physical), but not a subtle (e.g. sexual) forms of IPV. We conclude that there is a need to assess IPV situations giving key considerations for female empowerment and agency. This is critical for highlighting situations that may not be linear presentations of male-perpetration and female-victimization. Moreover, some communities have the tendency to demonstrate more gender-equitable attitudes regarding male perpetration of IPV more than others. These are key learnings that can inform the design and delivery of various interventions that seek to address IPV.

## Supporting information

S1 Fig Interview guide IDI. (DOCX)

S2 Fig Interview guide FGD. (DOCX)
